# Hydrogen sulfide: a gaseous signaling molecule modulates tissue homeostasis: implications in ophthalmic diseases

**DOI:** 10.1038/s41419-019-1525-1

**Published:** 2019-03-29

**Authors:** Yuyi Han, Qianwen Shang, Jin Yao, Yong Ji

**Affiliations:** 1 Department of Ophthalmology, Nanjing Medical University Affiliated Wuxi Second Hospital, Wuxi, China; 2 Institutes for Translational Medicine, Soochow University Medical College, Suzhou, China; 3 The Affiliated Eye Hospital of Nanjing Medical University, Nanjing, China; 4 The Fourth School of Clinical Medicine, Nanjing Medical University, Nanjing, China; 5 Key Laboratory of Cardiovascular and Cerebrovascular Medicine, Key Laboratory of Targeted Intervention of Cardiovascular Disease, Collaborative Innovation Center for Cardiovascular Disease Translational Medicine, Nanjing Medical University, Nanjing, China

## Abstract

Hydrogen sulfide (H_2_S) serves as a gasotransmitter in the regulation of organ development and maintenance of homeostasis in tissues. Its abnormal levels are associated with multiple human diseases, such as neurodegenerative disease, myocardial injury, and ophthalmic diseases. Excessive exposure to H_2_S could lead to cellular toxicity, orchestrate pathological process, and increase the risk of various diseases. Interestingly, under physiological status, H_2_S plays a critical role in maintaining cellular physiology and limiting damages to tissues. In mammalian species, the generation of H_2_S is catalyzed by cystathionine beta-synthase (CBS), cystathionine gamma-lyase (CSE), 3-mercapto-methylthio pyruvate aminotransferase (3MST) and cysteine aminotransferase (CAT). These enzymes are found inside the mammalian eyeballs at different locations. Their aberrant expression and the accumulation of substrates and intermediates can change the level of H_2_S by orders of magnitude, causing abnormal structures or functions in the eyes. Detailed investigations have demonstrated that H_2_S donors’ administration could regulate intraocular pressure, protect retinal cells, inhibit oxidative stress and alleviate inflammation by modulating the function of intra or extracellular proteins in ocular tissues. Thus, several slow-releasing H_2_S donors have been shown to be promising drugs for treating multiple diseases. In this review, we discuss the biological function of H_2_S metabolism and its application in ophthalmic diseases.

## Facts as indicated in the Instructions

- H_2_S is not only a poisonous gas, but also has critical role in maintaining homeostasis and functions of eye.
- H_2_S is endogenously generated and serves as a gaseous modulator in eye.
- H_2_S shows diverse effects on ocular tissues in both physiological or pathological situations, which are mostly influenced by its concentration.

## Open Questions

- The beneficial/toxic concentrations of H_2_S have not been established in different tissues.
- The most effective administrative route of H_2_S for different eye diseases needs to be determined.
- Drugs that can be co-administered with H_2_S for congenital ophthalmic diseases have not been determined.

## Introduction

Hydrogen sulfide (H_2_S) was identified by Carl Wilhelm Scheele through chemical analysis in the 17th century. However, it has long been believed that this gas emanated from the sewer system is related to a series of a special type of eye diseases occurred in sewer workers. This disease is associated with painful inflammation, secondary bacterial invasion and even blindness. Like nitric oxide (NO) and carbon monoxide, endogenously produced H_2_S is now known as another gaseous signaling molecule that affects the structure and function of proteins by participating in their short-lived covalent reactions^1^. This gasotransmitter can easily diffuse across cell membranes and does not need a specific mechanism for their degradation and reuptake. In human, the concentration of H_2_S in tissues can be at μM ranges for maintaining the physiological cellular functions. Its levels can differ according to age, tissues and measuring methods^2,3^. For example, the H_2_S concentration in the peripheral blood is generally 30–300 μM^4^, while the physiological concentration of H_2_S in the brain is up to three times of that in serum^5,6^. The H_2_S gas/water coefficient of distribution is 0.39, which can be affected by pH^2,7^. In comparison to healthy individuals, the H_2_S concentration in the serum of asthmatic patients can reach to 600 μM^8^.

The oxidation products of H_2_S include persulfide, sulfite, thiosulfate and sulfate^9^. When the concentrations of H_2_S in tissues or cells are high, H_2_S is considered as a toxic substance and its oxidation products may cause cytotoxic effects through inhibiting mitochondrial cytochrome C oxidase and disrupting cell energy production, leading to tissue inflammation or DNA damage^10^. However, when it is generated at physiological rates or at low concentrations, it has entirely different effects on biological processes such as cellular division, DNA repair and metabolism, modulation of protein kinase, regulation of cell cycle and organization of cytoskeletal framework^11^. Recent investigations have found that the potential regulatory role of H_2_S is to add cysteine, a thiol group in proteins (aka S-sulfhydration, or persulfide formation)^12^. This modification critically changes the physiological actions and pathological status of proteins in response to inflammation or oxidation by generating a –SSH group. The persulfides have better reactivity than corresponding thiols and can readily react with electrophiles. Persulfidation of proteins such as K_ATP_ contributes to various H_2_S-induced biochemical reactions^13^. When H_2_S is produced at low levels through enzymatical degradation of cysor homocysteine, it is critical in maintaining the functions of nervous system and vascular system^14,15^. Exogenously administrated H_2_S has been found to extend the lifespan of worms, relieve inflammation and promote reparation of injured tissues^16^. In views of the potential value of H_2_S in body systems and its presence in mammalian eyes^17^, this review focuses on the role of H_2_S in the common ophthalmic diseases and the underlying mechanisms, hoping to provide therapeutic strategy for ophthalmic diseases. Detailed analysis on the crosstalk between ocular tissues and H_2_S generation pathway will pave the road for understanding the pathogenesis of multiple ophthalmic diseases and optimize the application of H_2_S donor for treatment.

## Generation of H_2_S in ocular tissues

In mammalian cells, the generation of H_2_S is dependent on four major enzymes: cystathionine-γ-synthase (CSE), cystathionine-β-lyase (CBS), 3-mercapto-methylthio pyruvate aminotransferase (3MST) and cysteine aminotransferase (CAT)^18,19^. Generally, the generation of H_2_S relies on the desulfurization of cysteine or homocysteine by the two pyridoxial 5-phosphate (PLP)-dependent enzymes, CSE and CBS^7^. Of note, the distribution of these enzymes for H_2_S production shows tissue specificity. For example, CBS is the main enzyme for H_2_S generation in the central nervous system^20^. CSE is the major enzyme for H_2_S production in the vasculature system, liver, and kidney^21–23^. The presence of these H_2_S-productive enzymes are proved in ocular tissues, especially in the retina^24–26^. According to recent studies, H_2_S can also be produced from D-cysteine, catalyzed by D-Amino acid oxidase (DAO) and 3MST^27^.

**Fig. 1:**
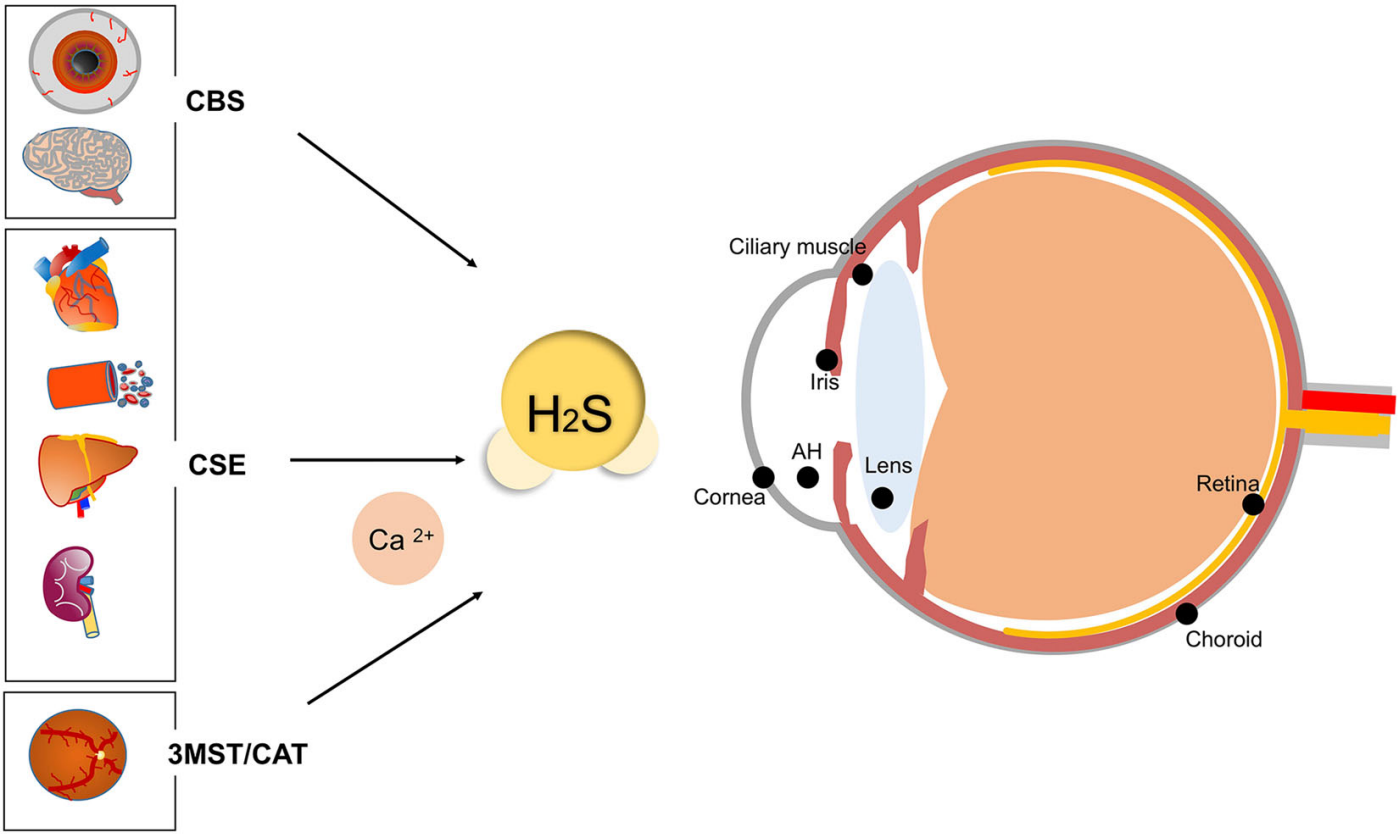
Generation of H_2_S. H_2_S generation is mainly controlled by four enzymes, CBS (major source of H_2_S production in central nervous system), CSE (major source of H_2_S production in vasculature system, liver and kidney), 3MST and CAT (major source of H_2_S production in retina). CSE and CAT are regulated by Ca^2+^. H_2_S is produced by these enzymes at steady-state low intracellular concentrations of Ca^2+^. Multiple ocular tissues showed the presence of endogenous H_2_S, including lens, iris, choroid, ciliary muscle, aqueous humor, cornea, and retina, except vitreous humor. The highest concentrations of endogenous H_2_S are detected in cornea and retina, of which the production differs in their major enzymes

Endogenously production of H_2_S is discovered in various tissues of bovine eye, including cornea, aqueous humor, iris, ciliary muscle, lens, choroid and retina, except vitreous humor. The highest production of endogenous H_2_S was detected in cornea and retina^17^. CBS is most highly expressed in the cornea, conjunctiva, and iris, while much lower amount be found in retina and optic nerve, relatively lower amount in lens, but absent in the vitreous humor. CBS expression remains high in anterior segments throughout the lifespan, and it has a trend of age-dependent increase in retina^24^. CSE is characterized in retina of amphibians and mammals, where its activity can be traced^25^. 3MST/CAT pathway is the dominating way to produce H_2_S in mammalian retina as both 3MST and CAT are located in the retinal neurons, which is increased at low concentrations of Ca^2+^ that achieved in brightness^26^. Deficiency of H_2_S or its substrates are found to be related to ectopialentis, myopic, cataract^28^, optic atrophy, and retinal detachment^29–31^ (Fig. 1).

### H_2_S and glaucoma

#### Reduction of intraocular pressure (IOP)

High IOP is the major cause for optic neuropathy in patients of glaucoma, which damages the retinal neurons and optic nerve heads^32^. Stable IOP depends on the balance of aqueous humor (AH) generation in the ciliary body and AH outflow in the chamber angle, especially in trabecular meshwork^33^. The outflow facility could be increased by cyclic adenosine monophosphate (cAMP) administration in anterior chamber for the maintenance of IOP^34^. H_2_S-releasing compounds could act on adenylyl cyclase and ATP-sensitive potassium channels (K_ATP_) channels in eyes, thus increase cAMP concentrations in porcine ocular anterior segments and help mediate the outflow of AH^35^. Ex vivo study has indicated that H_2_S participates in the phosphodiesterase (PDE) inhibition and enhancement of intramitochondrial cAMP levels, which stimulates protein kinase A (PKA) to instruct bioenergetic effects^36^. The inhibition of PDE activity by H_2_S is a relevant factor to cumulative cAMP and cyclic guanosine monophosphate (cGMP). Meanwhile, elevated intraocular cGMP level is related to reducing the trabecular meshwork cell volume and promoting outflow of AH^37^. H_2_S-producing donors such as GYY4137, are well-investigated for stabilizing IOP, as their administration upregulate the intraocular glutathione (GSH) expression with increased cGMP levels^38,39^.

H_2_S donors also work on anterior uvea to relax iris smooth muscles^40^ and thus lower IOP. On the other hand, norepinephrine released by intraocular degenerating sympathetic nerve terminals can cause a decrease in outflow facility with a subsequent elevated IOP in the long term, even though it may lead to an acute increase in outflow facility^41^. Increased levels of norepinephrine in AH during night is related to an increase rather than a decrease in IOP in rabbits^42^. H_2_S can reduce the release of norepinephrine from sympathetic nerves^43^, which contributes to stabilizing IOP.

#### Effect on ocular blood supply

Ischemia can cause glaucomatous damage accompany with or without an abnormal IOP. In vivo studies have revealed that inadequate blood supply can lead to optic nerve head atrophy and cell death in ganglions, which implies that abnormal ocular blood flow (OBF) necessarily affects metabolic processes to adapt to visual function needs^44^.

Several conflicting reports are published about the pharmacological reactions of H_2_S in vasculature of diverse organs in different species. It is reported that high concentrations of GYY4137 (1 mM) can significantly raise phenylephrine-induced tone in the ophthalmic arteries of rabbits^45^, but more evidences have proven that newly derived H_2_S donors exert vasodilator effects on pre-contracted posterior ciliary arteries (PCAs)^46,47^, which are crucial to OBF. Low concentrations of GYY4137 (100 nM–100 μM) may elicit relaxations in PCAs in the presence of phenylephrine induced tone via endogenous production of both prostanoids and H_2_S^47^. AP72 and AP67 show vasodilation effect on phenylephrine-induced PCAs in a concentration-dependent manner^46^. These effects are mainly dependent upon the action on K_ATP_ channels by H_2_S. Taken together, these studies have established the role of H_2_S in modulating the  OBF of glaucoma.

#### Protection on neurons

The major features of glaucoma include progressive cell death of retinal ganglions and optic nerve damage^48^,which are usually induced by loss of neurotrophic factors, intracellular and extracellular toxicity of glutamate, and neuro-inflammation^48–52^. In the nervous system, H_2_S functions as neurotransmitters^53^ and possesses the ability to inhibit apoptosis and degradation of neurons^54^. H_2_S produced by astrocytes acts as a synaptic modulator and causes excitation to nearby neurons by controlling calcium ion influx of astrocytes^55^. For eyes, in vitro experiments have demonstrated that addition of H_2_S donors to the culture system effectively inhibits the release of sympathetic neurotransmission from isolated bovine iris-ciliary bodies^56^, and inhibits amino acid neurotransmission in isolated bovine retina^57^, which is mediated by its action on the K_ATP_ channels or NO synthase.

H_2_S can not only enhances the N-methyl-D-aspartate (NMDA) receptor-mediated responses in physiological concentrations^20^, but also modulates the over-activated NMDA receptors via the cAMP axis^58–60^. Aberrant metabolism or signaling pathways of H_2_S are found in various neurodegenerative diseases, such as declined levels of H_2_S in Alzheimer’s patients^61^, impaired CSE transcription in Huntington’s disease^62^, depleted sulfhydration in Parkinson’s disease^63^, and increased H_2_S levels found in amyotrophic lateral sclerosis^64^. The fact that H_2_S modulates cell functions, protects neurons from apoptosis or oxidative stress are widely confirmed^65–67^. H_2_S is able to neutralize excess peroxynitrite (ONOO^−^) or other free radicals, to antagonize lipid peroxidation and oxidation of thiols, and to reverse mitochondrial dysfunction^7^. It works as an anti-oxidant for eliminating the excessive glutamate together with glutathione^68^, as well as activating K_ATP_ channels to combat oxidative glutamate toxicity^69^. H_2_S could inhibit the generation of reactive oxygen species (ROS)^70^ and ameliorate the toxic effect of hypochlorous acid (HOCl) generated from myeloperioxidase (MPO) catalysis, thereby exerting anti-oxidant effects and protecting neuronal cells from cellular chlorinative damage^71^. H_2_S presents anti-apoptotic effect on the SH-SY5Y cell line in low concentrations by preserving mitochondrial functions, which is referred to suppressing cytochrome oxidase C and opening the mitochondrial K_ATP_ channels^72^.

Referring to the anti-oxidant activity by H_2_S donors exerted on neurons, studies have found that H_2_S could increase the GSH concentration in neurons by enhancing the transporter of cysteine, cysteine/glutamate antiporter and γ-glutamyl cysteine synthetase (γ-GCS)^73,74^. γ-GCS and GSH synthetase act concertedly during the synthesis of GSH. Both enzymes can be regulated by Nrf2, which is also one potential targets of H_2_S^75^. The consequence of H_2_S-regulated Nrf2 pathway in neurons is to enhance the expression of glutathione-S-transferase (GST) and heme oxygenase (HO-1), the oxidative stress-related antioxidant enzymes^76^. ACS14 and ACS1, two donors of H_2_S, are confirmed to improve the intracellular GSH level and promote neuroprotective effects via opening K_ATP_ channels^77^.

**Fig. 2:**
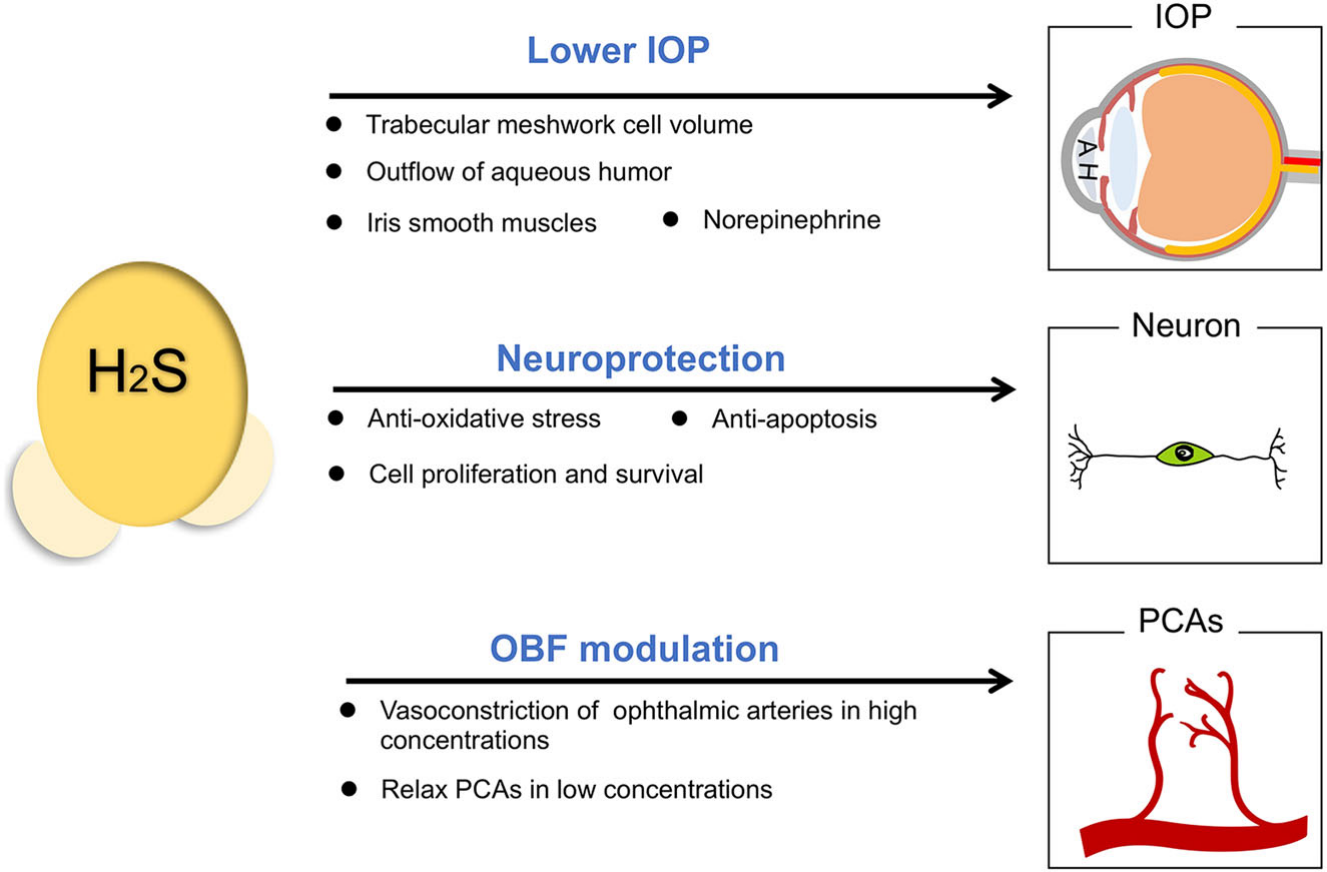
H_2_S and glaucoma. H_2_S donors lower IOP by the reduction of the trabecular meshwork cell volume, the promotion of AH outflow, the relaxation of iris smooth muscles and the decrease of norepinephrine release. H_2_S can relax PCAs to modulate the OBF, so that alleviates the glaucomatous damage induced by ischemia. It also attenuates cellular damage induced by oxidants and inhibits neuron apoptosis to protect RGCs

H_2_S could promote cell survival through effectively activating protein kinase C-α (PKC-α), inhibiting NF-κΒ signaling pathway, as well as upregulating Bcl-2 and X chromosome-linked inhibitor of apoptosis (XIAP) levels in RGC cells that pre-treated with glutamate (Glu) and buthionine sulfoximine (BSO)^76^. In comparison with glutamate treated RGC cells, addition of H_2_S enhances Akt phosphorylation and promotes cell viability in response to oxidative stress^76^. In a chronic ocular hypertension rat model, H_2_S is demonstrated to attenuate RGC apoptosis through balancing mitochondrial function, suppressing glial activation and downregulating the autophagy process^78^. Intracameral injection of NaHS to rats bearing glaucoma prohibits the loss of RGCs through recovering the levels of H_2_S in retina^79^. A long time release of H_2_S from GYY4137 combined with the in situ gel forming PLGA-based system, which lasts up to 72 h, has pointed to a great potential application in treating glaucoma^80^.(Fig. 2)

### H_2_S and diabetic retinopathy (DR)

#### Reduction of the effects of advanced glycation end products (AGEs) in DR

High glucose condition gives rise to the non-enzymatic condensation reaction between glucose and the amino terminus of protein, leads to the accumulation of AGEs’ macromolecule, which has close relationship with the occurrence of DR^81,82^. AGEs can crosslink intracellular proteins to disturb their functions, and interfere normal metabolic pathways such as ATP production. AGEs destroys the inner blood–retinal barrier (BRB) in eye with subsequent oxidative stress reactions and inflammation^83,84^.

H_2_S promotes galactose metabolism to reduce AGEs generation in neuronal cells and prohibit excessive oxidative stress^85^. Mechanistically, H_2_S reduces ROS production and lipid peroxidation, while enhancing the expression of superoxide dismutase (SOD) and glutathione peroxidase (GPX), two endogenous antioxidant enzymes^86^. In addition, H_2_S could reverse high glucose-induced increase in the expression of aldehyde oxidase 1 (AOX-1) and decrease in glutathione synthetase (GSS) level, ultimately to antagonize the AGEs-induced oxidative stress in cells^85^.

#### Inhibition of oxidative stress and inflammation

Although the toxicity of H_2_S accounts for the pathogenesis of multiple diseases, H_2_S possesses versatile anti-inflammatory effects in vivo or vitro.

High glucose levels disturb the electron transfer process of the cellular mitochondrial respiratory chain in diabetic patients, so that oxygen free radical O^−^ and superoxide can be easily generated^87^. Excessive O^−^ converts NO into ONOO^−^, which can irreversibly bind to cytochrome C and impair mitochondrial functions. In DR animal models, the enhanced level of intracellular oxygen species and its associated excessive lipid peroxidation can be suppressed by H_2_S^73,88^. One property of H_2_S in anti-inflammation is to scavenge the pro-inflammatory oxidants, such as ONOO^−^, HOCl, superoxide and hydrogen peroxide^71,89^. Besides, the pro-inflammatory response can be shifted to anti-inflammation by H_2_S donors, as demonstrated to decrease the levels of TNF-α, IL-8 and IFN-γ, while increasing the levels of cyclooxygenase (COX)-2 and eicosanoids^90^. Similarly, GYY4137 has been reported to inhibit LPS-induced production of inflammatory mediators by macrophages, and to upregulate the release of anti-inflammatory cytokine, IL-10^91^. Such regulation on inflammatory cytokine production can be attributed to the suppressive function of H_2_S on NF-κB activation^91,92^.

The animal models of DR have showed that hyperglycemia-induced leukostasis is related with cell apoptosis and retinal capillary occlusion^93^. The effect of resolving inflammation by H_2_S relies on its role in mediating macrophage phagocytosis^94^ and promoting the granulocytes survival through inhibition of p38 phosphorylation and caspase-3 cleavage^95^. It downregulates the expression of MPO in neutrophils, thereby alleviating some of their toxic actions^96^. Moreover, increased retinal expression of intercellular adhesion molecule-1 (ICAM-1) and leukocyte adhesion in vessels are observed in DR animal models^93^, but H_2_S could downregulate ICAM-1 expression in vascular endothelium under high glucose conditions^97^. The adhesion molecules, including lymphocyte function-associated antigen, P-selectin and ICAM-1, are indispensable in instructing immune cells to transmigrate across inflamed capillaries. Blockade of H_2_S synthetase abolishes the alleviation of inflammation, with increased adherence of leukocytes to vascular endothelium and their transmigration^98,99^.

Investigations on the regulation of H_2_S on myocardium in type 1 diabetic rat model has revealed that H_2_S interferes with the inducible NOS (iNOS)/NO system, inhibits iNOS activity and its catabolite mediated oxidative stress^100^. However, the anti-inflammatory function by H_2_S is not always achieved. In low dose, H_2_S donor inhibits the inflammatory response, while high doses of H_2_S donor achieves controversial results. Therefore, dosage is a switch to control the biphasic regulation of H_2_S donor on inflammation^101^, and the generation of H_2_S can be augmented by the appearance of inflammation^102^. The feedback mechanism of H_2_S in controlling the progression of inflammatory responses in DR remains unclear.

#### Protective effect on retinal neurons

During the pathological process of DR, neuron damage usually occurs earlier and accumulates into visible retinal vascular lesions^103^. ACS67, a H_2_S donor, can be used to prevent RGC apoptosis and reactive gliosis in Muller cells after ischemia-reperfusion or exposure to oxidative stress^104^. Also, administration of H_2_S donors recovers the expression of brain-derived neurotrophic factor (BDNF) and retinal synaptic vesicle protein in streptozotocin (STZ)-induced diabetic rats, indicating that H_2_S might block neuronal degeneration of retinal in diabetic patients^105^. The neuroprotective effect of H_2_S in retina is also related to its regulation on the intracellular GSH content^104^.

#### Multiple effects on retinal blood vessels

##### Dual role of BRB stability

The dysfunction of BRB is a primary cause of retinal vascular lesions during DR pathogenesis. In DR development, retinal ischemia and hypoxia stimulate the expression of hypoxia inducible factor (HIF-1α) and trigger subsequent vascular endothelial growth factor (VEGF) signaling activation. The HIF-1α-VEGF-VEGFR2 signaling pathway is responsible for diabetes-induced BRB dysfunction and excessive angiogenesis^106^. In vivo experiments have shown that the reduced BRB permeability and decreased acellular capillaries in retinas of STZ-induced diabetic rats after exogenous H_2_S treatment is accompanied by the reduction in VEGF content of vitreous and gene expression of VEGFR2, HIF-1α, as well as with increased expression of occludin^105^. Exogenous H_2_S administration is found to inhibit excessive deposition of laminin and collagen IVα3, in order to maintain the vascular integrity in the retinas of diabetic rats^107^. On the other hand, VEGF in intraocular tissues can stimulate endothelial cells to produce and release H_2_S^108^. At the onset of diabetes, H_2_S served as a protective factor against oxidative stress or nitrosative stress in the retina and vitreous humor, and it seems like H_2_S has a protective role on BRB in hyperglycemic condition. However, along with the progression of proliferative diabetic retinopathy (PDR), H_2_S may enhance the effect of VEGF on vascular endothelial cells, as well as the angiogenesis process^108–110^. Investigations on the level of H_2_S in the vitreous and plasma of PDR patients have revealed a much higher expression, indicating the potential effects of H_2_S in the pathogenesis of PDR^111^. As one of the main source to produce H_2_S in retina, 3-MST in hyperglycemic cells fail to convert 3-MP to H_2_S when the extracellular glucose concentration is elevated, and thus lost the ability of stimulating angiogenesis or cell proliferation, but the proangiogenic effect by exogenous H_2_S is not attenuated by hyperglycemia^112^. Moreover, the H_2_S-generating enzymes/H_2_S contributes to retinal neovascularization in ischemia-induced retinopathy^113^. These facts indicate that H_2_S may deteriorate retinal hemorrhage during the late stages of PDR.

##### Antithrombotic effect

Besides inflammation and apoptosis, platelet adhesion is also involved in diabetes-induced retinal endothelial dysfunction^93^. The platelet adhesion to the injured diabetic endothelium takes part in ischemia and inflammation, both coagulation and fibrinolytic cascades in the vitreous are identified in DR^114^. Blood platelets tend to adhere to the vascular endothelium of DR rather than normal vessels^115^, which is involved in retinal capillaries occlusion and microvascular damage. H_2_S plays a potential role in reducing platelet aggregation, cell adhesion, and coagulantion^116–119^, it exerts antithrombotic effect through upregulation of NO synthesis, hydrolysis of disulfide bonds and the reduction of the calcium concentration in platelets^119–121^.

##### Modulation of the retinal blood flow

**Fig. 3:**
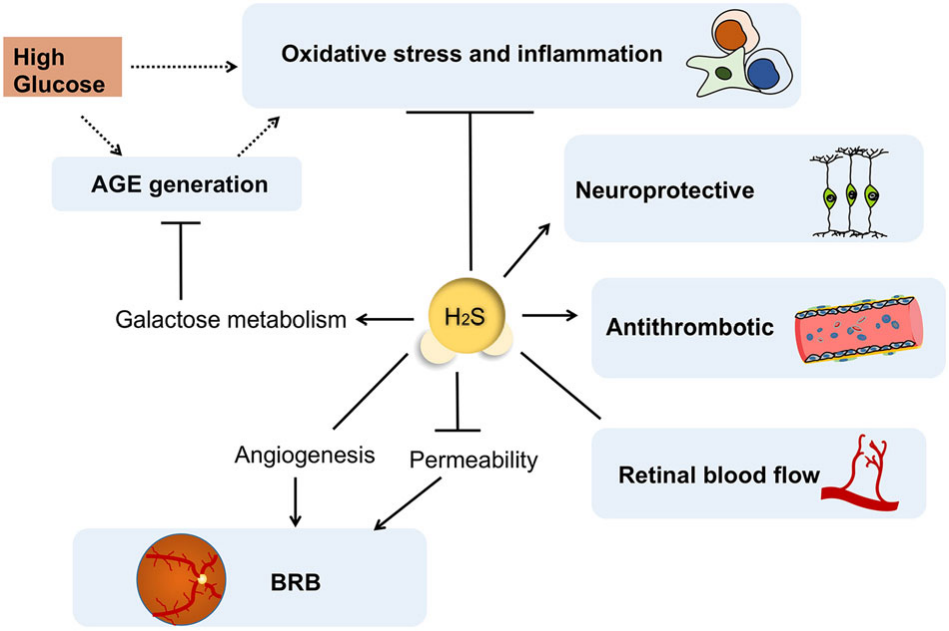
H_2_S and DR. H_2_S promotes galactose metabolism to reduce AGE generation in neuronal cells and antagonizes high glucose-induced oxidative stress and inflammation, as well as protects retinal neurons in diabetic patients. Although H_2_S can relief the BRB permeability of DR and exert an antithrombotic effect, it shows dual vascular effects on retinal vessels to modulate the retinal blood flow and participates in angiogenesis process

The altered retinal circulation of the diabetes is well documented, diabetic mice demonstrates reduced density of flowing deep vessels^122^. There may be both increased and decreased retinal blood flow in diabetic patients compared with healthy people, while no significant difference is observed in OBF between patients of nonproliferative DR and PDR^123^. Considering the possibility of ischemia and hypoxia induced by abnormal blood supply and vascular dysfunction, we notice that H_2_S has multiple effects on vessels. The application of H_2_S donors could protect blood vessels, regulate blood pressure and alleviate the inflammatory reactions in the vascular system^124^. H_2_S exhibits the dual vascular effects of vasoconstriction and vasodilation depends on the vascular district, the endothelium conditions, the H_2_S concentration and the method of precontraction^125^. Different from the increased cAMP production induced by H_2_S in brain cells^59^, H_2_S negatively modulates β-adrenoceptor function via suppressing the adenylyl cyclase activity in cardiac myocytes^126^. The adenyl cyclase/cAMP pathway is involved in H_2_S induced vasoconstriction^127^, but on the other hand, H_2_S can instruct vascular smooth muscle cells against excessive vascular contraction via affecting K_ATP_^9^. Moreover, H_2_S alleviates the contraction of vascular smooth muscle by reducing the concentration of intracellular calcium through acting on inositol 1,4,5-triphosphate receptor^128^. While its variable effects on vasculature were still being discussed, the increased cGMP level due to the PDE inhibition, the affected NO/cGMP pathway with activated endothelial nitric oxide synthase (eNOS) and COX-derived metabolic byproducts are all required for H_2_S-induced vasodilation^129,130^. In addition, the vasodilation induced by H_2_S is related to the promotion of prostaglandin generation^131^, angiotensin-converting enzyme inhibition^102^, as well as modulating the viability of anion exchangers to control intra-cellular pH value^132^. All these findings imply that H_2_S contributes to regulating retinal blood flow and is involved in the DR pathogenesis. (Fig. 3)

### H_2_S and retinal degeneration

#### Modulation and protection of retinal neurons

Several retinal degenerative diseases, such as retinitis pigmentosa (RP) and age-related macular degeneration (AMD), are associated with aberrant function of retinal pigment epithelium (RPE) and photoreceptor cells, which are crucial to maintain accurate visual sense^133^.

One of the RPE features is the apical and basal membranes, where the apical parts envelop photoreceptor cell outer segment (POS) to remove and degrade them through phagocytosis^134^. With a circadian rhythm, the phagocytosis of POS distal tips is always triggered by light^135^. Disturbance of RPE phagocytic function leads to POS accumulation and inevitable photoreceptor degeneration. The deficiency of CBS activity and the accumulation of homocysteine in the retina may lead to abnormal RPE structure and functions, bringing about the development of AMD-like features^136^.

Another feature of RPE cells is melanogenesis for absorbing excess light and protect photoreceptors. Melanin dispersion toward the apical microvilli of the RPE correlates positively with the intracellular level of cAMP, while light suppresses cAMP synthesis in retina of mice^137^. It has been shown that cAMP stimulates melatonin synthesis^138^ and elevated cAMP levels with its signaling system influences RPE migration^139^. Increased cAMP in the subretinal space can lead to entry of cAMP into RPE cells via organic anion transporters with consequent triggering of dark-specific physiological responses, the nonderivatized cAMP can activate pigment granule aggregation in isolated RPE sheets^140^. It is reported that H_2_S donors and its substrate could produce a time and dose-dependent increase in cAMP concentrations in rat RPE cells^141^, the process of which involves K_ATP_ channels and the enzymes of CSE and CBS^141^.

The metabolic cascade of photoreceptor signal transduction is mediated by cGMP that synthesized by guanylyl cyclases in retinal neurons. The response is triggered when photopigments absorb light, with subsequent degradation of cGMP by PDE^142^. Activities of cAMP-hydrolyzing and cGMP-hydrolyzing have been detected in homogenates of cultured pigment epithelia from rats^143^. It is reported that cGMP stimulates the absorption of subretinal fluid by activating the RPE cell pump^144^, which is consistent with the fact of decreased cGMP concentration in the retinal detachment cases^142^. As mentioned above, H_2_S participates in the inhibition of PDE activity and induction of cyclic nucleotides, and at least three forms of PDEs are present in human RPE cells^145^, we infer that the cumulative cAMP or cGMP instructed by H_2_S may help maintain physiological functions of RPE and photoreceptors.

Administration of H_2_S contributes to protecting retinal neurons from light-induced degeneration^26^. Chronic sustained light-induced damages in the macular area cause degeneration of RPE and photoreceptor cells. Long-term excessive light exposure can induce damage or death of photoreceptor cells by oxidative stress and intracellular calcium overload^146^. Calcium in relatively low level can activate the 3MST/CAT enzymes to produce H_2_S. In turn, H_2_S can prevent Ca^2+^ influx in the photoreceptor cells by activating V-ATPase in horizontal cells and maintain the balance of intracellular calcium, so that H_2_S protects photoreceptor cells from retinal cell apoptosis and oxidative stress^26^. However, the regulation of Ca^2+^ and the cytoprotective effect of endogenous H_2_S may fail when photoreceptor cells are under excessive light exposure.

#### Potential in stem cell transplantation therapy

The RPE cells can modulate photoreceptor differentiation and retinal progenitor cells, which may play a role in the regulation of the retinal stem cell niche^147^. Transplantation of stem cell-derived RPE is proven to be effective in reversing retinal degeneration such as AMD^148,149^. MSCs are multipotent stem cells with self-renewal abilities, immunoregulatory functions and multiple lineage differentiation potentials. In vitro expanded MSCs have been widely applied to treat many tissue injury, such as myocardial infarction^150^, skin wound^151^, organ transplantation^152^, autoimmune diseases^153^, and retina injuries^154^. MSCs express CBS and CSE, and produce H_2_S^155^, with a positive feedback on the proliferation and survival of MCSs^156^. Studies have found that increased endogenous H_2_S level can block the hypoxia and serum deprivation-induced MSC apoptosis^157^, both the ERKs signaling pathways and the Akt signaling pathway are involved in the promotion of H_2_S on stem cell proliferation^158,159^. NaHS can prolong the survival of bone marrow mesenchymal stem cells (BMMSCs) and enhance their therapeutic effects for ischemic injury, also can improve the blood vessel integrity and prompt angiogenesis, with the upregulation of BDNF and VEGF expression^160^. In its regulation on stem cell differentiation, H_2_S is likely to affect neurogenesis by directly regulating Ca^2+^ channels^161^, to initiate endothelial progenitor cell function and to enhance the angiogenesis process of wound sites in type 2 diabetic patients^162^.

**Fig. 4:**
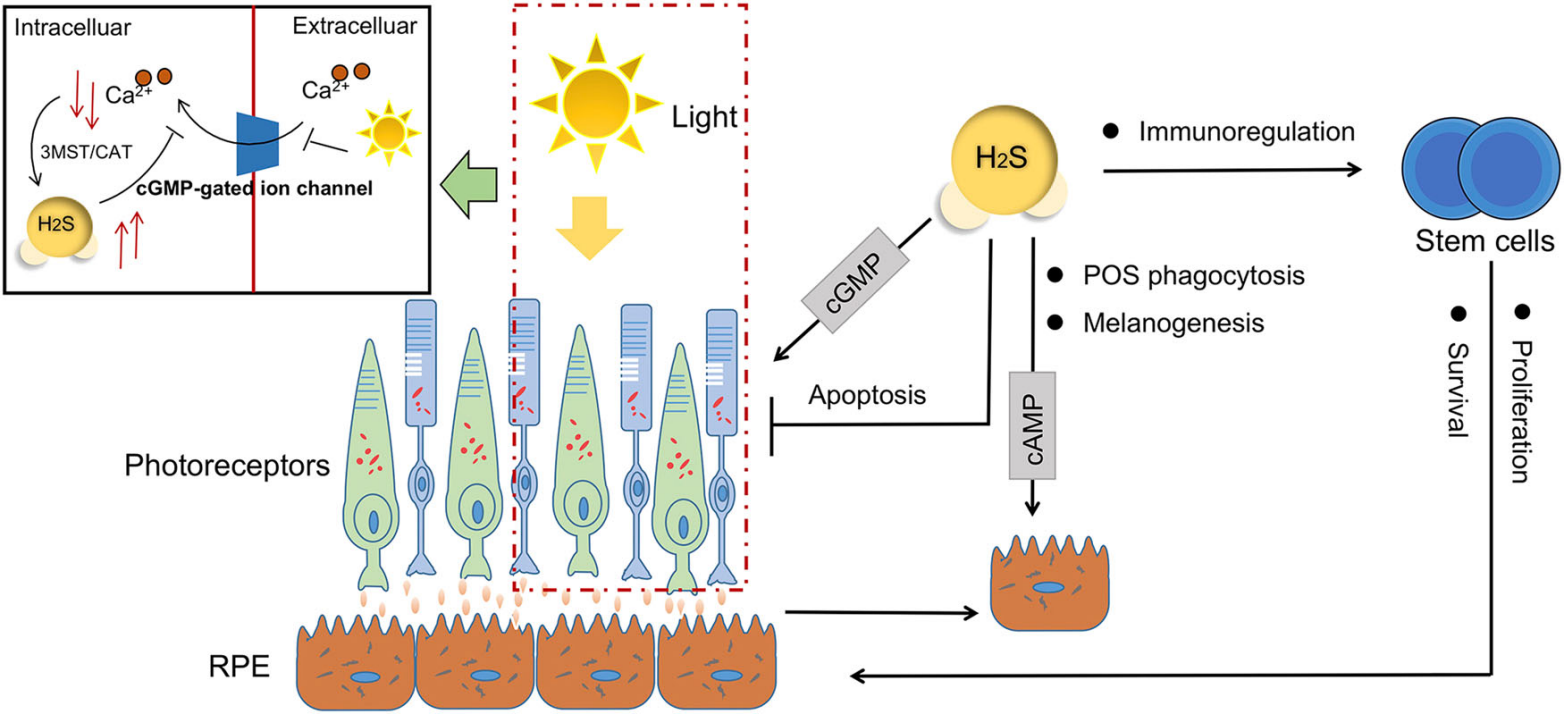
H_2_S and retinal degeneration. H_2_S and its productive enzymes are involved in the phagocytosis of POS and melanogenesis in RPE cells, as well as in photoreceptor signal transduction. Long-term excessive light exposure induces photoreceptor cell damage which is related to intracellular calcium overload. When the retinal photoreceptor cells are exposed under high intensity illumination, the cGMP-gated ion channels in membrane are shut down, with a cascade activity resulted in a relative low level of intracellular calcium. Such status facilitates H_2_S generation catalyzed by 3MST/CAT enzymes, subsequently suppress Ca^2+^ influx by activating V-ATPase. Exogenous H_2_S reduces the number of apoptotic retinal neurons after excessive light irradiation. Another approach to treating retinal degeneration disease is the stem cell-derived RPE-based therapy. Studies have found that H_2_S affects the immunoregulatory function of MSCs, and can enhance the proliferation and survival of the stem cells, thus improve their ability in tissue repair

Also, H_2_S is featured as one of the potential molecule for immunoregulation by MSCs. Deficiency of H_2_S attenuates the immunosuppressive function of MSCs on colitis in vivo, while supplementation of NaHS can restore the impaired therapeutic effects^163^. By the way, clinical H_2_S treatment is expected to improve long-term allograft survival in conjunction with immunosuppression for its positive effects on promoting organ survival against cold ischemia reperfusion injury^164^. Considering that NaHS pretreatment can enhance stem cells proliferation, promote the survival of therapeutically used stem cells and tissue cells via increased antioxidant defense^165^, H_2_S may be useful for the regeneration of retinal photoreceptors and RPE cells via transplantation strategies (Fig. 4).

## Perspectives

Investigations in the past decade have provided new insights into the function of H_2_S during tissue damage and repair. In addition to its toxic effects, H_2_S is found to reduce intraocular pressure, inhibit inflammation and oxidative stress, promote stem cell-based regeneration, and restore the retinal microcirculation homeostasis. However, the exact therapeutic and pathological concentration of H_2_S remains elusive. Recently, novel H_2_S releasing drugs such as ATB-346 and ATB-352, have shown the efficacy in treating digestive diseases, with the promising application potential in various eye diseases. Due to the complexity of the BRB and the special anatomical structures of the eyes, the administrative routes of H_2_S should be carefully considered. Further investigation in this exciting field is expected to provide detailed information for better understanding the function of H_2_S in different types of eye diseases, and to design more effective and safe approaches for H_2_S application in clinical settings.
